# Evolutionary and Functional Relationships of the *dha* Regulon by Genomic Context Analysis

**DOI:** 10.1371/journal.pone.0150772

**Published:** 2016-03-03

**Authors:** Marinalva Martins-Pinheiro, Wanessa C. Lima, Huma Asif, Cláudio A. Oller, Carlos F. M. Menck

**Affiliations:** 1 Dept of Microbiology, Institute of Biomedical Sciences, University of São Paulo, São Paulo, 05508–900, Brazil; 2 Dept. of Chemical Engineering, Polytechnic School, University of São Paulo, São Paulo, Brazil; 3 Dept. of Pharmacology, University of Heidelberg, Heidelberg, D-69120, Germany; University of Illinois, UNITED STATES

## Abstract

3-hydroxypropionaldehyde (3-HPA) and 1,3-propanediol (1,3-PD) are subproducts of glycerol degradation and of economical interest as they are used for polymers synthesis, such as polyesters and polyurethanes. Some few characterized bacterial species (mostly from Firmicutes and Gamma-proteobacteria groups) are able to catabolize these monomers from glycerol using the gene products from the *dha* regulon. To expand our knowledge and direct further experimental studies on the regulon and related genes for the anaerobic glycerol metabolism, an extensive genomic screening was performed to identify the presence of the *dha* genes in fully sequenced prokaryotic genomes. Interestingly, this work shows that although only few bacteria species are known to produce 3-HPA or 1,3-PD, the incomplete regulon is found in more than 100 prokaryotic genomes. However, the complete pathway is found only in a few dozen species belonging to five different taxonomic groups, including one Archaea species, *Halalkalicoccus jeotgali*. Phylogenetic analysis and conservation of both gene synteny and primary sequence similarity reinforce the idea that these genes have a common origin and were possibly acquired by lateral gene transfer (LGT). Besides the evolutionary aspect, the identification of homologs from several different organisms may predict potential alternative targets for faster or more efficient biological synthesis of 3-HPA or 1,3-PD.

## Introduction

The rapidly growing biodiesel industry is responsible for the generation of an excessive amount of crude glycerol, a by-product of biodiesel production from plant oils or animal fats. Glycerol is also an important carbon source for bacteria and yeast. Under aerobic and anaerobic conditions, these organisms can use glycerol for metabolic energy acquisition, as a regulator of the redox potential and for the recycling of inorganic phosphate in the cell [1]. Moreover, under anaerobic or microaerobic conditions some bacteria are capable of converting glycerol to 3-hydroxypropionaldehyde (3-HPA) and 1,3-propanediol (1,3-PD) [2].

1,3-PD is an important monomer of economical interest for industrial use, with numerous applications in the synthesis of polymers and other organic chemicals. The known bacterial producers of 1,3-PD from glycerol include three genera belonging to the Gamma-proteobacteria (*Citrobacter* [3–5], *Enterobacter* [6] and *Klebsiella* [7, 8]), and three from the Firmicutes group (*Clostridium* [9–11], *Lactobacilli* [12], and *Trichococcus* [13]). Three other Proteobacteria genera (*Ilyobacter* [14], *Pelobacter* [15] and *Anaerovibrio* [16]) are also known to produce small amounts of 1,3-PD from glycerol. Up to now, among these microorganisms, *Clostridia butyricum* and *Klebsiella pneumoniae* are the most important natural producers and therefore of great biotechnological importance for the production of this monomer. However, efforts to improve the production of this monomer in these and other species (e.g. through application of metabolic engineering) met only with limited success [2, 17, 18].

**Fig 1:**
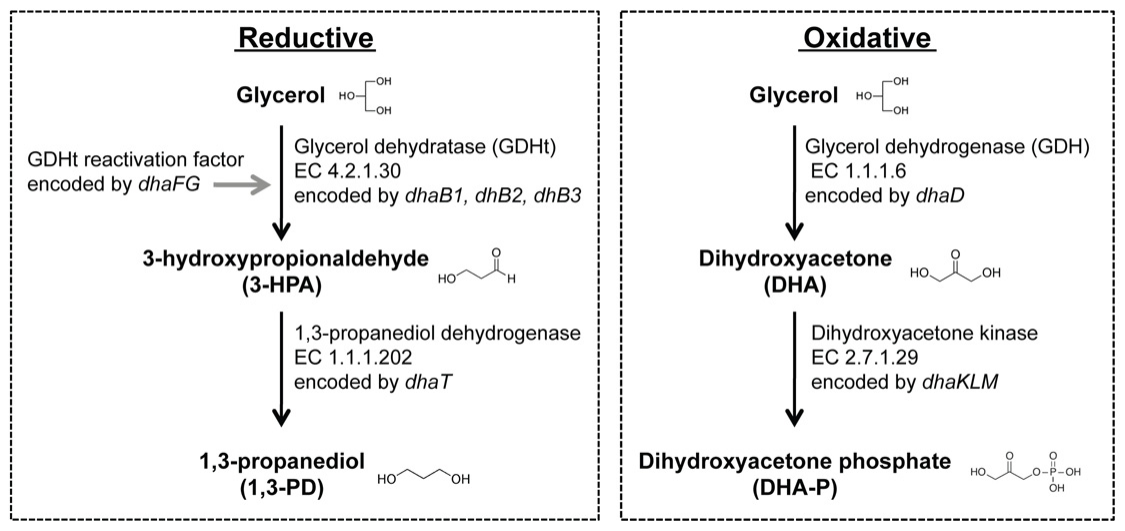
Glycerol assimilation through the fermentative pathway, showing the oxidative and reductive pathways. In the oxidative route, glycerol is converted in dihydroxyacetone (DHA) and dihydroxyacetone phosphate (DHA-P) by the products of *dhaD* and *dhaMKL* genes. In the reductive branch, glycerol is reduced to 1,3-PD by the successive action of glycerol dehydratase (GDHt) (encoded by three *dhaB* genes) and 1,3 propanediol oxidoreductase (encoded by *dhaT* gene). The genes refer to those found in *K*. *pneumoniae*.

Biologically, glycerol is metabolized in a dismutation process involving two branches, reductive and oxidative pathways (carried out by enzymes coded by the regulon *dha* in the *Klebsiella* genus) (Fig 1). The oxidative route is performed by the glycerol dehydrogenase (encoded by *dhaD*) with the generation of NADH, which transforms the glycerol to dihydroxyacetone (DHA). DHA is then transformed by the products of *dhaMKL* genes to dihydroxyacetone phosphate (DHA-P), and DHA-P enters the glycolytic pathway to form pyruvate. In the reductive branch, glycerol can be dehydrated to 3-HPA by the B12-vitamin dependent glycerol dehydratase (GDHt) (encoded by *dhaB1*, *dhaB*2 and *dhaB*3 genes in *K*. *pneumoniae*), or its isozyme dioldehydratase (*pduCDE* genes in *Citrobacter* and *Lactobacillus* species) or by the B12-independent GDHt (encoded by *dhaB1* and *dhaB*2 in *Clostridium butyricum*). The inactive GDHt is substrate of a reactivase, which is encoded by two genes (*dhaF* and *dhaG*). Then, 3-HPA is reduced to 1,3-PD by 1,3 propanediol oxidoreductase (encoded by *dhaT* gene in *K*. *pneumoniae*) or NADPH-dependent oxidoreductase (encoded by *yqhd* gene in *E*. *coli*) [19–21].

Although the production of 3-HPA and 1,3-PD by bacteria is a well-studied process [21, 22], not much is known about its presence in other bacterial groups outside Proteobacteria and Firmicutes, or even in Archaea. The availability of thousands of completely sequenced microbial genomes provides a huge source for investigation of this regulon in distinct organisms. A comprehensive overview of the *dha* regulon in *Klebsiella* and *Citrobacter* is available [23], but an increasing number of genomes have been sequenced since then. As a result, the opportunity to find new and interesting *dha*-related genes among a variety of sequenced bacterial and archaeal genomes is enormous. In this context, genomic analysis may be a useful tool to optimize the production in both natural producers and heterologous hosts.

In the present work, we describe a large-scale inventory of the *dha* regulon and related genes in bacterial and archaeal completely sequenced genomes, and focus on some selected organisms for a detailed molecular evolutionary analysis.

## Results and Discussion

### Large-scale identification of *dha* genes in completely sequenced genomes

**Fig 2:**
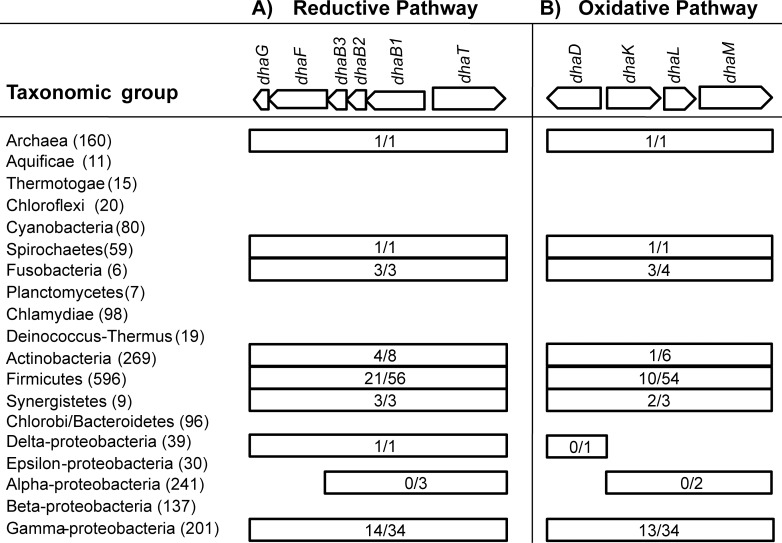
Distribution of *dha* genes in completely sequenced Bacteria and Archaea genomes. On top, the genomic arrangement of *dha* genes present in *Klebsiella pneumoniae* is depicted; arrows indicate the direction of transcription. In parenthesis by the side of each taxonomic group, the number of total genomes analyzed within each group is given. Taxonomic groups in which *dha* genes were identified are depicted by rectangular boxes; the numbers inside indicate the number of genomes with “the complete pathway /at least one gene”, either reductive (A) or oxidative (B).

From more than 2,000 complete prokaryotic genomes analyzed (Fig 2), only 111 possess at least part of the *dha* regulon (S1 Table), and belong to several bacterial taxonomic phyla (Actinobacteria, Firmicutes, Fusobacteria, Proteobacteria, Synergistetes and Spirochaetes) and one Archaea representative (Fig 2). However, the complete regulon is present in only a very reduced number of genomes (S2 Table).

The complete reductive pathway–comprising the three *dhaB* subunits (or the isofunctional *pduCDE* genes), *dhaFG* and *dhaT* (or the isofunctional *yqhd* gene)–is present in a large number of genomes from the Gamma-proteobacteria (14 genomes) and Firmicutes groups (21), Fusobacteria (3), Spirochaetes (1), Delta-proteobacteria (1), Synergistetes (3) and Actinobacteria (4) (S1 Table and Fig 2). Surprisingly, one Archaea genome (*H*. *jeotgali*) contains the complete regulon. As stated before, the reduction of 3-HPA to 1,3-PD is carried out by the NADH-dependent 1,3-PD oxidoreductase (encoded by *dhaT* gene), or through the NADPH-dependent oxidoreductase (encoded by *yqhd* gene in *E coli*). The absence of both genes in some of the genomes analyzed (notably in the three species of Alpha-proteobacteria; for complete list, refer to S1 Table), but in which the *dhaB* gene is present, suggests that these organisms might possess other enzymes able to make this conversion or, alternatively, that 3-HPA is used as preferred substrate for alternative reactions.

The complete oxidative pathway, comprising *dhaDKLM* genes, on the other hand, presents a more restricted distribution: only in one Spirochaetes and Actinobacteria, two Synergistetes, thirteen Gamma-Proteobacteria genomes (three of them belonging to the well-characterized *Klebsiella* genus), ten Firmicutes, three Fusobacteria and one Archaea species (Fig 2). As it is the case for other organisms investigated here, the Archaea *H*. *jeotgali* also possesses *dhaDKLM* orthologs but no *dhaR* regulator gene. So, in this work, we considered organisms as having the complete regulon even in the absence of *dhaR* gene.

The presence of the two potentially complete pathways in some Firmicutes and Gamma-proteobacteria species was already known, as the two-best characterized species in genetics and biochemistry terms (*C*. *butyricum* and *K*. *pneumoniae*) belong to these groups. However, the presence of the complete regulon on the Archaea *H*. *jeotgali* is surprising, and strengthens the validity of a genomic approach to find new targets for further biochemical characterization. The organisms with both complete pathways include Spirochaetes (1), Synergistetes (2), Fusobacteria (3), Archaea (1), Firmicutes (6) and Gamma-proteobacteria (9) (S2 Table).

Given the patchy distribution of the *dha* genes in the taxonomic groups here analyzed, we decided to study their possible evolutionary origin, with a special focus on *Brachyspira intermedia* (Spirochaetes), *Desulfatibaccilum alkenivorans* (Delta-proteobacteria), *H*. *jeotgali* (Archaea), *Ilyobacter polytropus* (Fusobacteria), *Hyphomicrobium sp*., *Mesorhizobium loti* and *Mesorhizobium opportunistum* (Alpha-proteobacteria). These organisms were chosen because they are the unique species with *dha* genes within a large group of bacteria (Fig 2).

### Genomic organization of the *dha* regulon

The genes coding for the reductive pathway are normally assembled in two operons: one formed by the genes coding for GDHt (*dhaB1*, *dhaB2*, *dhaB3*) and its reactivation factor (*dhaFG*), and the second constituted by the gene *dhaT* (1,3-PD oxidoreductase) (Fig 2). Similar configuration is found for the genes coding for the oxidative pathway: the first operon comprises *dhaR* and *dhaD* and, the second, *dhaK*, *dhaL* and *dhaM* genes. In the natural producers, the genes of each pathway are naturally controlled by two different promoters and transcribed in different directions [23], but they are normally clustered together (Fig 2).

**Fig 3:**
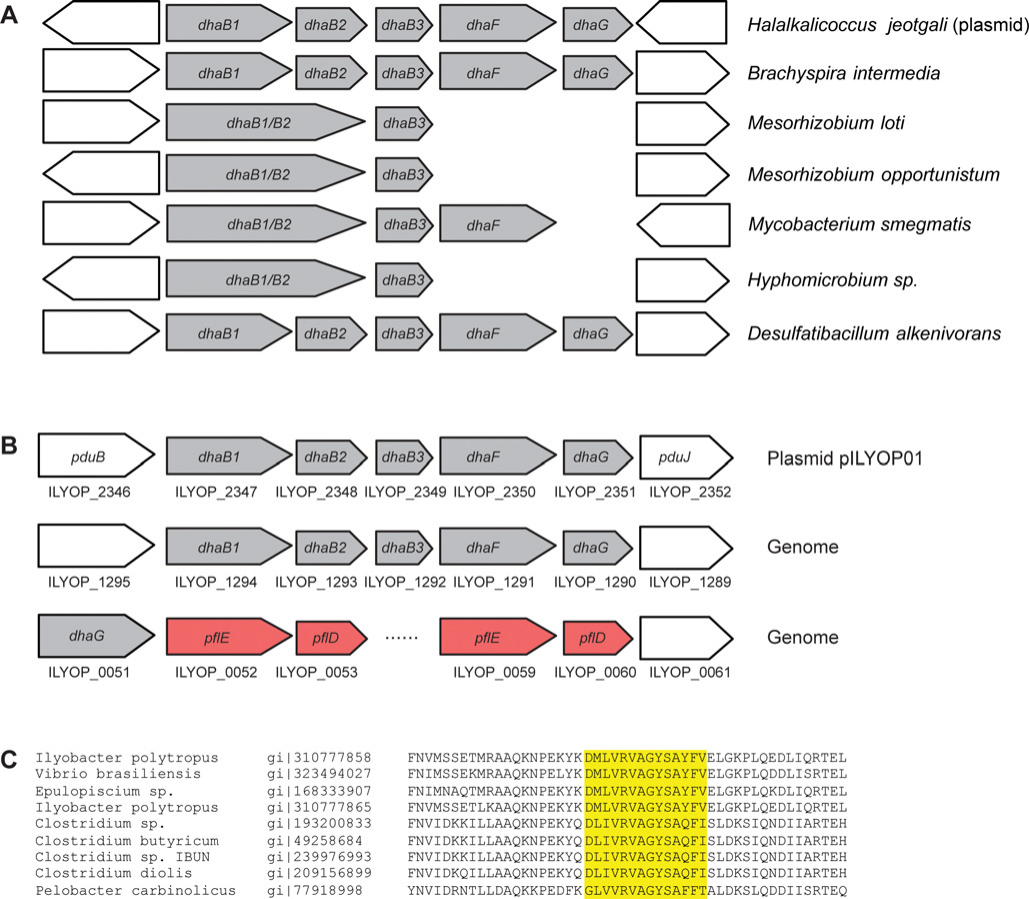
Genomic organization of the reductive pathway. (A) Orthologous *dha* genes (in grey) are represented by block arrows showing their position and orientation (out of scale). Gene arrangement is conserved in the seven organisms shown. *Mesorhizobium*, *Mycobacterium* and *Hyphomicrobium* have fused *dhaB1* and *dhaB2* genes. (B) Fusobacterium *I*. *polytropus* contains two *dha* regulons: genes coding for GDHt B12-dependent and its reactivation factor (grey arrows) are found in the plasmid or in the genome; and those coding for B12-independent (red arrows) are present in the genome. (C) Partial sequence alignment of the B12-independent glycerol dehydratase large subunit showing a highly conserved region corresponding to a glycyl radical domain.

The seven species here analyzed present a conserved syntenic architecture in respect to gene order and orientation of the reductive pathway, as verified for the natural producer species. This syntenic conservation may indicate that LGT events have shaped this genomic configuration (Fig 3A and 3B). Regarding the oxidative pathway, gene order is not conserved in these organisms.

It is interesting to note that all bacterial species described as able to metabolize glycerol to 1,3-PD are anaerobic or anaerobic facultative, consistent with the necessary anaerobic or micro aerobic conditions for the production of 1,3-PD [24]. Therefore, the description of novel orthologs in aerobic organisms may have implications for the production of 1,3-PD in aerobic conditions. This is the case for five of the seven species focused in this work, which are reported as aerobic [25–28] or, in the case of *Brachyspira sp*., oxygen-tolerated anaerobe [29] (Table 1).

**Table 1:** Genes identified for the reductive and oxidative pathway in the anaerobic metabolism of glycerol. Comparison of genes from *Klebsiella pneumoniae* to *B*.*intermedia*, *D*. *alkenivorans*, *H*. *jeotgali*, *Hyphomicrobium sp*., *I*. *polytropus*, *M*. *loti* and *M*. *opportunistum*.

			Species	*Halalkalicoccus jeotgali*	*Brachyspiraintermedia*	*Mesorhizobium loti*	*Mesorhizobium opportunistum*	*Hyphomicrobium sp*.	*Desulfatibacillum alkenivorans*	*Ilyobacter polytropus*
			**Phylum (Class)**	Archaea	Spirochaetes	Alpha-proteobacteria	Alpha-proteobacteria	Alpha-proteobacteria	Delta-proteobacteria	Fusobacteria
			**Oxygen requirement**	Aerobic	Oxygen-tolerant anaerobe	Aerobic	Aerobic	Aerobic	Anaerobic	Anaerobic
			**ORF number** *	HacjB3_	Bint_	mll	Mesop_	HYPMC_	Dalk_	Ilyop_
**Reductive route**	**Glycerol to 3-HPA**	Glycerol dehydratase B-12 independent	*dhaB1*							0053 (61%)0060 (61%)
			*dhaB2*							0052 (58%)0059 (58%)
		Glycerol dehydratase B-12dependent or its isozyme dioldehydratase	*dhaB1*	**15946** (80%)	1617 (85%)	6722 (67%)	2992 (66%)	3786 (66%)	4999 (79%)	**2347** (83%)1294 (87%)
			*dhaB2*	**15941** (65%)	1616 (78%)	absent	absent	absent	4998 (71%)	**2348** (77%)1293 (81%)
			*dhaB3*	**15936** (67%)	1615 (75%)	6721 (60%)	2991 (60%)	3787 (66%)	4997 (67%)	**2349**(74%)1292 (76%)
		Glycerol dehydratase reactivation factor	*dhaF*	**15931**(73%)	1614 (77%)	absent	absent	absent	4996 (72%)	**2350** (76%)1291 (74%)
			*dhaG*	**15926**(47%)	1613 (52%)	absent	absent	absent	4995 (51%)	**2351** (51%)1290 (48%)
	**3-HPA to 1,3 PD**	1,3-propanediol oxidoreductase	*dhaT*	03495 (48%)	0018 (53%)	3070 (53%)	1883 (52%)	absent	1481 (59%)	1297 (86%)0504 (74%)
			*yqhd*	03495 (44%)	2514 (55%)	3070 (39%)	1883 (39%)	absent	4698 (69%)	0978 (71%)
**Oxidative Route**	**Glycerol to DHA**	Glycerol dehydrogenase	*dhaD*	10515 (62%)	1863 (47%)	absent	1883 (43%)	absent	0861 (61%)	10777 (73%)
	**DHA to DHA-P**	Didydroxyacetone kinase	*dhaK*	07520 (61%)	2220 (66%)	5289 (60%)	0220 (61%)	absent	absent	0851 (64%)
			*dhaL*	07525 (53%)	2221 (60%)	5296 (49%)	0215 (55%)	absent	absent	0850 (57%)
			*dhaM*	07530 (59%)	2222 (49%)	absent	0216 (57%)	absent	absent	0849 (54%)

* ORF number refers to the name given to each gene during the sequencing project.

ORFs in bold and underlined are present in plasmids. Numbers in parenthesis indicate similarity percentage to *K*. *pneumoniae* genes or, in the case of glycerol dehydratase B12-independent genes, to *Clostridium butyricum*.

### Primary structure conservation of *dha* genes

Conservation of gene order across distant phylogenetic groups is considered an evidence of LGT [30, 31], but the degree of conservation of the primary sequence among distant may also be taken as evidence for transfer events.

Concerning the *dhaB* genes, they present a high degree of primary sequence similarity to *Klebsiella* or *Clostridium* genes (above 60%; Table 1). However, *H*. *jeotgali* DhaB large subunit possesses a N-terminal extension of 18 amino acid residues, and in *M*. *loti* a gene fusion resulted in a single gene coding for the large and medium subunits (*dhaB1* and *dhaB*2) (Fig 3A). This particular domain arrangement has been reported for a few species, notably *Mycobacterium* and *Mesorhizobium* [32], and in the present work it was also found in the genome of the alpha-proteobacteria *Hyphomicrobium* sp.

Liu and coworkers [32] observed that the active site of fused genes coding for glycerol dehydratase in *M*. *loti* and *Mycobacterium* is slightly different from those of other organisms in which this enzyme is encoded by different genes. In the same work, the authors hypothesize a better catalytic activity for this protein as a consequence of a more efficient reactivation process, as already verified in fused genes obtained through mutagenesis [33]. Possible similar properties for this protein in *Mesorhizobium*, *Mycobacterium* and *Hyphomicrobium* may suggest that they would be a good model for studies aiming at enhancing the production of 3-HPA and 1,3-PD. Otherwise, the absence of genes coding for the reactivation factor in these genera led us to hypothesize that reactivation process may not exist or be carried out by a different route in these bacteria.

Some organisms possess, apart from the phosphoenolpyruvate-dependent dihydroxyacetone kinase (DhaKII), an ATP-dependent dihydroxyacetone kinase (DhaKI) (S1 Table). This kinase consists of two domains homologous to DhaK and DhaL. However, in *K*. *pneumoniae* [34], Dhak I has no significant contribution for the conversion DHA to DHA-P.

In the majority of the organisms, the GDHt enzyme requires cobalamin (vitamin B12) as a cofactor, exceptions being the form present in *Clostridium butyricum* [35], and more recently also identified in *Clostridium methylpentosum*, *Pelobacter carbinolicus*, *Ruminococcus* and *Salmonella typhimurium* [32]. In addition, we identified in this work that *Vibrio brasiliensis* (genome not yet completely sequenced) and *Ilyobacter polytropus* also present the B12-independent GDHt. Interestingly, *I*. *polytropus* presents a duplication of both genes coding for B12-independent and B12-dependent GDHt. The genes coding for the B12-independent form appears in tandem in its genomic DNA, and the latter type with one copy residing in the genome, and the other in the plasmid pILYOP01 (Fig 3B and S1 Table). Curiously, even with this gene redundancy, it has not been reported a significant production of 3-HPA or 1,3-PD in *I*. *polytropus* [14]. Comparison of the amino acid sequence of the two putative B12-independent GDHts from *I*. *polytropus* with the well characterized protein from *Clostridium butyricum* shows that they share the conserved glycine radical domain, present in all B12-independent GDHts [32] (Fig 3C).

These structural features observed for GDH in *M*. *loti*, *Mycobacterium* and *Hyphomicrobium* through gene fusion merit to be investigated. Gene fusion may arise by frameshift mutations leading to the loss of good Shine-Dalgarno sequences, and a possible selective advantage needs functional characterization. Other interesting point is that *M*. *loti*, *M*. *opportunistum*, *Hyphomicrobium* [25–28] and *H*. *jeotgali* are aerobic organisms, and the possibility of this regulon to be functional may be of great value, since all natural producers of 3-HPA and 1,3-PD are obligatory or facultative anaerobes.

### Evolutionary history of the *dha* genes revealed by phylogenetic analysis

On visual inspection of the *dha* regulon in several microbial genomes, it was found that the gene order showed significant synteny at least for the reductive pathway. This fact, combined with the patchy genic distribution, reinforces the hypothesis that these genes might have been laterally acquired.

**Fig 4:**
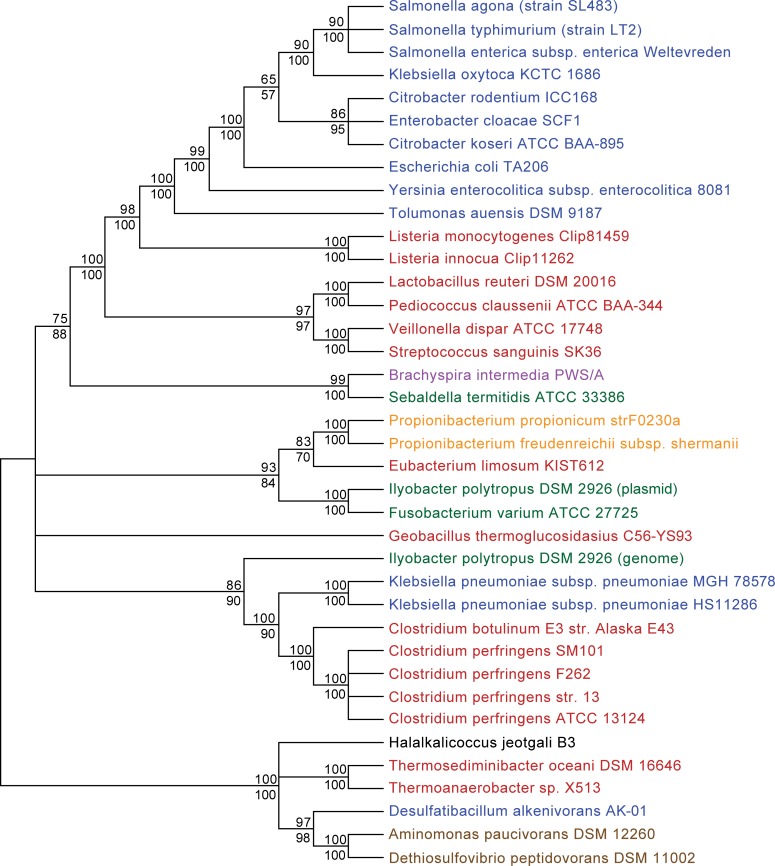
Evolutionary history of *dha* genes. Maximum-likelihood phylogenetic tree of concatenated *dhaB1*, *dhaB2*, *dhaB3*, *dhaF* and *dhaG* genes. Numbers at the nodes indicate the percentage of bootstrap support (upper values for the ML tree and lower values for the NJ tree; only number above 50% are shown). Nodes with less than 50% bootstrap support are condensed. Colors depict different taxonomic groups: Proteobacteria (blue), Firmicutes (red), Spirochaetes (purple), Fusobacteria (green), Actinobacteria (orange), Synergistetes (brown).

To further analyze the evolutionary story of the regulon, we performed phylogenetic reconstructions using the protein sequences of the five genes coding for the reductive pathway (*dhaB1*, *dhaB2*, *dhaB3*, *dhaF* and *dhaG*) (Fig 4). The resulting phylogenetic tree shows a patchy distribution among different bacterial lineages, with a complex evolutionary history. Two major monophyletic branches (with high bootstrap support) can be distinguished: one containing most of the Gamma-proteobacteria and Firmicutes species, and another with the Synergistetes, the Archaea *H*. *jeotgali*, the Delta-proteobacteria *D*. *alkenivorans*, and two Firmicutes. However, the relationship between the other major Bacterial groups (Actinobacteria, Fusobacteria, Spirochaetes) is not fully resolved (politomies represent nodes in which bootstrap values were below 50%). Interestingly, *Clostridium* and *Klebsiella pneumoniae* (but not *K*. *oxytoca*) species, and the Fusobacteria *I*. *polytropus* (genomic genes) form a robust monophyletic group, while the plasmidial copy *I*. *polytropus* genes clusters with the other Fusobacteria species.

This tree topology is conserved independently of the alignment algorithm or evolutionary model used for phylogenetic reconstruction (data not shown). Likewise, phylogenetic reconstruction using only the 3 subunits of the *dhaB* genes shows a very similar topology, with minor modifications on the basal nodes (some unresolved relationships that were supported in the 5-gene tree) (S1 Fig). Most notably, Alpha-Proteobacteria species form a basal group at the root of the tree, and the relationship between Synergistetes, Archaea and Delta-proteobacteria is poorly resolved.

The patchy distribution of the *dha* genes in major Bacterial groups, the high conservation in primary sequence, and the strong phylogenetic support for clusters of unrelated, distant species indicate that these genes were acquired independently, at different evolutionary steps, by lateral gene transfer events.

## Concluding Remarks

Microbial conversion of renewable resources to 3-HPA and 1,3-PD is a safe and environmentally friendly route to give an appropriated end to the glycerol, considering that this pathway can replace the traditional petrochemical and other chemical methods of synthesis [2]. But, up to now, strains capable of doing so have only been found in bacteria belonging to few phylogenetic groups (as Firmicutes and Gamma-proteobacteria) and with yields far from the expected for industrial scale [2, 36]. Therefore, it is interesting to investigate other prokaryote species that have the potential to produce these monomers, by analyzing their gene content and how the genes are distributed in the genome.

Although most of the *in silico* inferences must be confirmed and tested by experimentation, this work provides a profile of the genes responsible for the anaerobic metabolism of glycerol in several aerobic bacterial species and even in one Archaea, contributing to the understanding of the distribution of this regulon and providing new insights into the taxonomic distribution and evolutionary history of the *dha* genes. Besides, it also provides a useful framework for further functional investigation, as the results indicate a high conservation of the primary gene sequence.

The widespread distribution of part of the *dha* regulon (the reductive pathway, to convert glycerol into 3-HPA) suggests that it may be of far wider importance than it has been previously recognized. Likewise, the enzymes of this pathway may have other functions not related to 3-HPA or 1,3 PD production in the absence of the oxidative pathway. Although we could not identify the complete oxidative pathway for metabolizing glycerol in the majority of organisms here analyzed, it is possible that these organisms metabolize glycerol requiring an additional carbon source. For example, in some strains of lactobacilli, this additional carbon source is used to generate the reducing equivalents necessary to complement the reductive step [9]; this sugar-to-glycerol co-fermentation is used in *Lactobacillus* species due to the absence of specific enzymes of the oxidative pathway [37, 38].

Our predictions may help the characterization of positive targets for biological synthesis of 3-HPA and 1,3-PD with higher efficiency and employing less effort. A successful example of applied genetic information obtained through sequence analysis may be given by the isoenzyme of 1,3-PD of *E*. *coli* coded by the *yqhd* gene. Co-expression of this gene and *dhaT* improves the production of 1,3-PD in *K*. *pneumoniae* [39] whilst the presence of the isoenzyme in *E*. *coli* is not a guarantee of 1,3-PD production. Exploring the genetic basis of the *dha* regulon might improve the possibility of characterizing new genes in organisms that do not naturally ferment glycerol but possess part of the regulon.

In summary, our study reveals an extensive (and previously undescribed) sharing of *dha* regulon genes among unrelated bacteria groups, suggesting that these genomes might represent an important reservoir to be explored.

## Methodology

BLAST similarity searches using *dha* genes from *Klebsiella pneumoniae* as seed (S1 Table) were conducted on the NCBI database of complete prokaryotic genomes. Candidate genes were thus confirmed both by reverse sequence similarity searches (BlastP) and domain analysis.

Protein sequences of a set of three (*dhaB1*, *dhaB2*, *dhaB3*) or five concatenated genes (*dhaB1*, *dhaB2*, *dhaB3*, *dhaF* and *dhaG)* derived from a diverse group of organisms were aligned using ClustalW, MUSCLE or T-Coffee algorithms [40, 41, 42]. The alignment was then refined in order to remove regions that were hyper variable or with gaps. Phylogenetic trees were generated using MEGA 6.0 [43]. Genetic distances were computed using the Jones-Taylor-Thornton algorithm and Neighbor-Joining (NJ) was used to generate distance-based phylogenetic trees. Maximum-likelihood (ML) phylogenetic estimates were obtained from the concatenated data with the Le_Gascuel_2008 model [44, 45]. Sequence evolution model was selected using the “find best model option” in MEGA 6.0. Bootstrap assessment of tree topology with 100 replicates was performed to find the support and stability for the inferred clades. Similar topologies were found for the three alignment algorithms and two phylogenetic methods employed; the trees displayed in Fig 4 and S1 Fig correspond to the maximum-likelihood topologies (with bootstrap values for both ML and NJ trees shown), and alignment generated by MUSCLE. The organisms and the accession codes of genes investigated in the phylogenetic analysis are shown in S3 Table.

## Supporting Information

S1 Fig Evolutionary history of *dha* genes. Maximum-likelihood phylogenetic tree of concatenated *dhaB1*, *dhaB2* and *dhaB3* genes. Numbers at the nodes indicate the percentage of bootstrap support (upper values for the ML tree and lower values for the NJ tree; only number above 50% are shown). Nodes with less than 50% bootstrap support are condensed. Colors depict different taxonomic groups: Proteobacteria (blue), Firmicutes (red), Spirochaetes (purple), Fusobacteria (green), Actinobacteria (orange), Synergistetes (brown).
(TIF)

S1 Table *dha* regulon genes in different groups of Bacteria and Archaea. Species with completely sequenced genome in which at least one *dha* gene could be identified are represented in the table. The organisms are listed in alphabetic order within the group. Each sequence is identified by the corresponding accession code. For easier visualization, genes from the reductive pathway are depicted in blue, and red for the oxidative pathway (only the genes considered as part of the complete regulon are shaded).
(XLSX)

S2 Table List of organisms with complete *dha* regulon. The organisms are listed in alphabetic order within the group with the corresponding gene accession code.
(XLSX)

S3 Table List of species used in the phylogenetic analysis. The organisms are listed in alphabetic order with the corresponding gene accession codes.
(PDF)

## References

[1] Dills SS, Apperson A, Schmidt MR, Saier MH. Carbohydrate transport in bacteria. Microbiol Rev. 1980;44(3):385–418. 6999324 10.1128/mr.44.3.385-418.1980 PMC373186

[2] Saxena RK, Anand P, Saran S, Isar J. Microbial production of 1,3-propanediol: Recent developments and emerging opportunities. Biotechnol Adv. 2009;27(6):895–913. 10.1016/j.biotechadv.2009.07.003 19664701

[3] Barbirato F, Grivet JP, Soucaille P, Bories A. 3-Hydroxypropionaldehyde, an inhibitory metabolite of glycerol fermentation to 1,3-propanediol by enterobacterial species. Appl Environ Microbiol. 1996;62(4):1448–1451. 8919810 10.1128/aem.62.4.1448-1451.1996 PMC167915

[4] Seifert C, Bowien S, Gottschalk G, Daniel R. Identification and expression of the genes and purification and characterization of the gene products involved in reactivation of coenzyme B12-dependent glycerol dehydratase of Citrobacter freundii. Eur J Biochem. 2001;268(8):2369–2378. 11298756 10.1046/j.1432-1327.2001.02123.x

[5] Ainala SK, Ashok S, Ko Y, Park S. Glycerol assimilation and production of 1,3-propanediol by Citrobacter amalonaticus Y19. Appl Microbiol Biotechnol. 2013;97(11):5001–5011. 10.1007/s00253-013-4726-z 23377788

[6] Barbirato F, Soucaille P, Bories A. Physiologic Mechanisms Involved in Accumulation of 3-Hydroxypropionaldehyde during Fermentation of Glycerol by Enterobacter agglomerans. Appl Environ Microbiol. 1996;62(12):4405–4409. 16535461 10.1128/aem.62.12.4405-4409.1996 PMC1388999

[7] Yang G, Tian J, Li J. Fermentation of 1,3-propanediol by a lactate deficient mutant of Klebsiella oxytoca under microaerobic conditions. Appl Microbiol Biotechnol. 2007;73(5):1017–1024. 16960737 10.1007/s00253-006-0563-7

[8] Mu Y, Teng H, Zhang DJ, Wang W, Xiu ZL. Microbial production of 1,3-propanediol by Klebsiella pneumoniae using crude glycerol from biodiesel preparations. Biotechnol Lett. 2006;28(21):1755–1759. 16900328 10.1007/s10529-006-9154-z

[9] Biebl H, Menzel K, Zeng AP, Deckwer WD. Microbial production of 1,3-propanediol. Appl Microbiol Biotechnol. 1999;52(3):289–297. 10531640 10.1007/s002530051523

[10] Luers F, Seyfried M, Daniel R, Gottschalk G. Glycerol conversion to 1,3-propanediol by Clostridium pasteurianum: cloning and expression of the gene encoding 1,3-propanediol dehydrogenase. FEMS Microbiol Lett. 1997;154(2):337–345. 9311132 10.1111/j.1574-6968.1997.tb12665.x

[11] González-Pajuelo M, Meynial-Salles I, Mendes F, Soucaille P, Vasconcelos I. Microbial conversion of glycerol to 1,3-propanediol: physiological comparison of a natural producer, Clostridium butyricum VPI 3266, and an engineered strain, Clostridium acetobutylicum DG1(pSPD5). Appl Environ Microbiol. 2006;72(1):96–101. 16391030 10.1128/AEM.72.1.96-101.2006 PMC1352194

[12] Talarico TL, Dobrogosz WJ. Purification and Characterization of Glycerol Dehydratase from Lactobacillus reuteri. Appl Environ Microbiol. 1990;56(4):1195–1197. 16348166 10.1128/aem.56.4.1195-1197.1990 PMC184372

[13] van Gelder AH, Aydin R, Alves MM, Stams AJ. 1,3-Propanediol production from glycerol by a newly isolated Trichococcus strain. Microb Biotechnol. 2012;5(4):573–578. 10.1111/j.1751-7915.2011.00318.x 22117537 PMC3815333

[14] Stieb M, Schink B. A new 3-hydroxybutyrate fermenting anaerobe, Ilyobacter polytropus, gen nov, sp nov, possessing various fermentation pathways. Arch Microbiol. 1984;140(2):139–146.

[15] Schink B, Stieb M. Fermentative degradation of polyethylene glycol by a strictly anaerobic, gram-negative, nonsporeforming bacterium, Pelobacter venetianus sp. nov. Appl Environ Microbiol. 1983;45(6):1905–1913. 6881964 10.1128/aem.45.6.1905-1913.1983 PMC242557

[16] Ouattara AS, Traore AS, Garcia JL.Characterization of Anaerovibrio burknabensis sp nov, a lactate-fermentin bacterium isolated from rice field soils. Int J Syst Evol Microbiol.1992;42: 390–397.

[17] Wilkens E, Ringel AK, Hortig D, Willke T, Vorlop KD. High-level production of 1,3-propanediol from crude glycerol by Clostridium butyricum AKR102a. Appl Microbiol Biotechnol. 2012;93(3):1057–1063. 10.1007/s00253-011-3595-6 21972131

[18] Oh BR, Seo JW, Heo SY, Luo LH, Hong WK, Park DH, et al Efficient production of 1,3-propanediol from glycerol upon constitutive expression of the 1,3-propanediol oxidoreductase gene in engineered Klebsiella pneumoniae with elimination of by-product formation. Bioprocess Biosyst Eng. 2013;36(6):757–763. 10.1007/s00449-013-0901-y 23361186

[19] Wang W, Sun J, Hartlep M, Deckwer WD, Zeng AP. Combined use of proteomic analysis and enzyme activity assays for metabolic pathway analysis of glycerol fermentation by Klebsiella pneumoniae. Biotechnol Bioeng. 2003;83(5):525–536. 12827694 10.1002/bit.10701

[20] Seo JW, Seo MY, Oh BR, Heo SY, Baek JO, Rairakhwada D, et al Identification and utilization of a 1,3-propanediol oxidoreductase isoenzyme for production of 1,3-propanediol from glycerol in Klebsiella pneumoniae. Appl Microbiol Biotechnol. 2010;85(3):659–666. 10.1007/s00253-009-2123-4 19626321

[21] Celińska E. Debottlenecking the 1,3-propanediol pathway by metabolic engineering. Biotechnol Adv. 2010;28(4):519–530. 10.1016/j.biotechadv.2010.03.003 20362657

[22] Liu H, Xu Y, Zheng Z, Liu D. 1,3-Propanediol and its copolymers: research, development and industrialization. Biotechnol J. 2010;5(11):1137–1148. 10.1002/biot.201000140 21058316

[23] Sun J, van den Heuvel J, Soucaille P, Qu Y, Zeng AP. Comparative genomic analysis of dha regulon and related genes for anaerobic glycerol metabolism in bacteria. Biotechnol Prog. 2003;19(2):263–272. 12675558 10.1021/bp025739m

[24] Chen X, Zhang DJ, Qi WT, Gao SJ, Xiu ZL, Xu P. Microbial fed-batch production of 1,3-propanediol by Klebsiella pneumoniae under micro-aerobic conditions. Appl Microbiol Biotechnol. 2003;63(2):143–146. 12908084 10.1007/s00253-003-1369-5

[25] Kaneko T, Nakamura Y, Sato S, Asamizu E, Kato T, Sasamoto S, et al Complete genome structure of the nitrogen-fixing symbiotic bacterium Mesorhizobium loti. DNA Res. 2000;7(6):331–338. 11214968 10.1093/dnares/7.6.331

[26] Reeve W, Nandasena K, Yates R, Tiwari R, O'Hara G, Ninawi M, et al Complete genome sequence of Mesorhizobium opportunistum type strain WSM2075(T.). Stand Genomic Sci. 2013;9(2):294–303. 10.4056/sigs.4538264 24976886 PMC4062634

[27] Roh SW, Nam YD, Nam SH, Choi SH, Park HS, Bae JW. Complete genome sequence of Halalkalicoccus jeotgali B3(T), an extremely halophilic archaeon. J Bacteriol. 2010;192(17):4528–4529. 10.1128/JB.00663-10 20601480 PMC2937367

[28] Moore RL. The biology of Hyphomicrobium and other prosthecate, budding bacteria. Annu Rev Microbiol. 1981;35:567–594. 6170249 10.1146/annurev.mi.35.100181.003031

[29] Håfström T, Jansson DS, Segerman B. Complete genome sequence of Brachyspira intermedia reveals unique genomic features in Brachyspira species and phage-mediated horizontal gene transfer. BMC Genomics. 2011;12:395 10.1186/1471-2164-12-395 21816042 PMC3163572

[30] Ochman H, Jones IB. Evolutionary dynamics of full genome content in Escherichia coli. EMBO J. 2000;19(24):6637–6643. 11118198 10.1093/emboj/19.24.6637 PMC305890

[31] Kurland CG, Canback B, Berg OG. Horizontal gene transfer: a critical view. Proc Natl Acad Sci U S A. 2003;100(17):9658–9662. 12902542 10.1073/pnas.1632870100 PMC187805

[32] Liu Y, Gallo AA, Bajpai RK, Chistoserdov A, Nelson A, Segura L, et al The diversity and molecular modelling analysis of B-12 and B-12-independent glycerol dehydratases. Int J Bioinform Res Appl. 2010;6(5):484–507. 21224206 10.1504/IJBRA.2010.037988

[33] Wang XD, Zhang XE, Guo YC, Zhang ZP, Cao ZA, Zhou YF. Characterization of glycerol dehydratase expressed by fusing its alpha- and beta-subunits. Biotechnol Lett. 2009;31(5):711–717. 10.1007/s10529-009-9911-x 19152074

[34] Wei D, Wang M, Jiang B, Shi J, Hao J. Role of dihydroxyacetone kinases I and II in the dha regulon of Klebsiella pneumoniae. J Biotechnol. 2014;177C:13–19. 10.1016/j.jbiotec.2014.02.011 24583287

[35] Raynaud C, Sarçabal P, Meynial-Salles I, Croux C, Soucaille P. Molecular characterization of the 1,3-propanediol (1,3-PD) operon of Clostridium butyricum. Proc Natl Acad Sci U S A. 2003;100(9):5010–5015. 12704244 10.1073/pnas.0734105100 PMC154289

[36] Nakamura CE, Whited GM. Metabolic engineering for the microbial production of 1,3-propanediol. Curr Opin Biotechnol. 2003;14(5):454–459. 14580573 10.1016/j.copbio.2003.08.005

[37] Veiga da Cunha M, Foster MA. Sugar-glycerol cofermentations in lactobacilli: the fate of lactate. J Bacteriol. 1992;174(3):1013–1019. 1732191 10.1128/jb.174.3.1013-1019.1992 PMC206182

[38] Veiga-da-Cunha M, Foster MA. 1,3-Propanediol:NAD+ oxidoreductases of Lactobacillus brevis and Lactobacillus buchneri. Appl Environ Microbiol. 1992;58(6):2005–2010. 1622279 10.1128/aem.58.6.2005-2010.1992 PMC195718

[39] Zhuge B, Zhang C, Fang H, Zhuge J, Permaul K. Expression of 1,3-propanediol oxidoreductase and its isoenzyme in Klebsiella pneumoniae for bioconversion of glycerol into 1,3-propanediol. Appl Microbiol Biotechnol. 2010;87(6):2177–2184. 10.1007/s00253-010-2678-0 20499228

[40] Thompson JD, Gibson TJ, Plewniak F, Jeanmougin F, Higgins DG. The CLUSTAL_X windows interface: flexible strategies for multiple sequence alignment aided by quality analysis tools. Nucleic Acids Res. 1997;25(24):4876–4882. 9396791 10.1093/nar/25.24.4876 PMC147148

[41] Edgar RC.MUSCLE: multiple sequence alignment with high accuracy and high throughput Nucleic Acids Res. 2004;32(5):1792–1797. 15034147 10.1093/nar/gkh340 PMC390337

[42] Notredame C, Higgins DG, Heringa J. T-Coffee: A novel method for fast and accurate multiple sequence alignment. J Mol Biol. 2000;302(1):205–217. 10964570 10.1006/jmbi.2000.4042

[43] Tamura K, Stecher G, Peterson D, Filipski A, Kumar S. MEGA6: Molecular Evolutionary Genetics Analysis version 6.0. Mol Biol Evol. 2013;30(12):2725–2729. 10.1093/molbev/mst197 24132122 PMC3840312

[44] Le SQ, Gascuel O. An improved general amino acid replacement matrix. Mol Biol Evol. 2008;25(7):1307–1320. 10.1093/molbev/msn067 18367465

[45] Le SQ, Lartillot N, Gascuel O. Phylogenetic mixture models for proteins. Philos Trans R Soc Lond B Biol Sci. 2008;363(1512):3965–3976. 10.1098/rstb.2008.0180 18852096 PMC2607422

