# Supplementary figures and images for: Evolutionary and Functional Relationships of the dha Regulon by Genomic Context Analysis
Source: PLoS One. 2016 Mar 3;11(3):e0150772. doi: 10.1371/journal.pone.0150772 (PMC4777399; doi:10.1371/journal.pone.0150772)

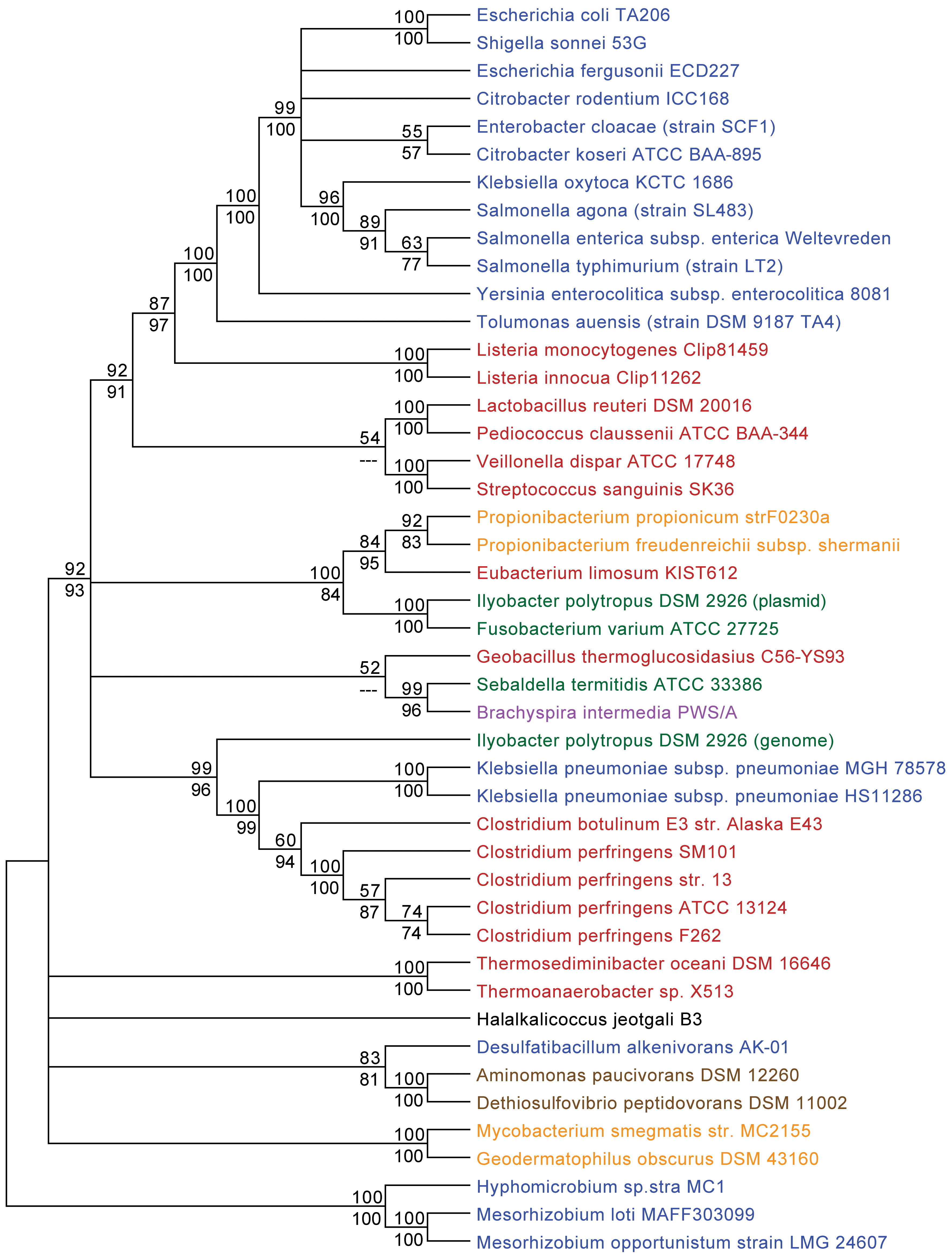

Supplement: S1 Fig — Maximum-likelihood phylogenetic tree of concatenated dhaB1, dhaB2 and dhaB3 genes. Numbers at the nodes indicate the percentage of bootstrap support (upper values for the ML tree and lower values for the NJ tree; only number above 50% are shown). Nodes with less than 50% bootstrap support are condensed. Colors depict different taxonomic groups: Proteobacteria (blue), Firmicutes (red), Spirochaetes (purple), Fusobacteria (green), Actinobacteria (orange), Synergistetes (brown). (TIF) [file pone.0150772.s001.tif]
