# Supplementary material for: Evolutionary and Functional Relationships of the dha Regulon by Genomic Context Analysis
Source: PLoS One. 2016 Mar 3;11(3):e0150772. doi: 10.1371/journal.pone.0150772 (PMC4777399; doi:10.1371/journal.pone.0150772)
Supplement: S3 Table — The organisms are listed in alphabetic order with the corresponding gene accession codes. (PDF) [file pone.0150772.s004.pdf]

Table S3: Species included in the phylogenetic analysis. The organisms are listed in alphabetic order.

Species	Division	*Genes*				
		dhaB1	*dhaB2*	*dhaB3*	*dhaF*	*dhaG*
Aminomonas paucivorans DSM 12260	Synergistetes	ZP_07738567	ZP_07738568	ZP_07738569	ZP_07738570	ZP_07738571
Brachyspira intermedia	Spirochaetes	AEM22236	AEM22235	AEM22234	AEM22233	AEM22232
Citrobacter koseri ATCC BAA-895	Gamma-proteobacteria	YP_001452384	YP_001452383	YP_001452382	YP_001452381	YP_001452380
Citrobacter rodentium ICC168	Gamma-proteobacteria	YP_003365676	YP_003365677	YP_003365678	YP_003365679	YP_003365680
Clostridium botulinum B str. Eklund 17B	Firmicutes	YP_001886294	YP_001886293	YP_001886292	YP_001886291	YP_001886290
Clostridium botulinum E3 str. Alaska E43	Firmicutes	ACD51295.1	ACD53765.1	ACD54032.1	ACD53335.1	ACD52785.1
Clostridium perfringens ATCC 13124	Firmicutes	ABG84836.1	ABG84161.1	ABG83167.1	ABG82533.1	ABG82537.1
Clostridium perfringens F262	Firmicutes	EIA17496	EIA17497.1	EIA17498.1	EIA17499.1	EIA17501.1
Clostridium perfringens SM101	Firmicutes	ABG86532.1	ABG86176.1	ABG86725.1	ABG87321.1	ABG86503.1
Clostridium perfringens str. 13	Firmicutes	NP_561845	NP_561846	NP_561847	NP_561848	NP_561849
Desulfatibacillum alkenivorans AK-01	Delta-proteobacteria	YP_002434138	YP_002434137	YP_002434136	YP_002434135	YP_002434134
Dethiosulfovibrio peptidovorans DSM 11002	Synergistetes	ZP_06392821	ZP_06392822	ZP_06392823	ZP_06392824	ZP_06392825
Enterobacter cloacae (strain SCF1)	Gamma-proteobacteria	ADO48014.1	ADO48013.1	ADO48012.1	ADO48011.1	ADO48004.1
Escherichia coli TA206	Gamma-proteobacteria	EGI28325	EGI28326	ZP_08359032	EGI28328	EGI28330
Escherichia fergusonii ECD227	Gamma-proteobacteria	EGC95113.1	EGC95114.1	EGC95115.1	EGC95116.1	EGC95599.1
Eubacterium limosum KIST612	Firmicutes	YP_003961988	YP_003961987	YP_003961986	YP_003961985	YP_003961984
Fusobacterium varium ATCC 27725	Fusobacteria	ZP_08693582	ZP_08693583	ZP_08693584	ZP_08693585	ZP_08693586
Geobacillus thermoglucosidasius C56-YS93	Firmicutes	YP_004587968	YP_004587969	YP_004587970	YP_004587971	YP_004587972
Geodermatophilus obscurus DSM 43160	Actinobacteria	YP_003407458	YP_003407459	YP_003407457	YP_003407460	YP_003407461
Halalkalicoccus jeotgali B3	Euryarchaeota	YP_003738338	YP_003738337	YP_003738336	YP_003738335	YP_003738334
Hyphomicrobium sp.	Alpha-proteobacteria	YP_004677562	Fused with *dhab1*	YP_004677563	absent	absent
Ilyobacter polytropus DSM 2926	Fusobacteria	ADO84107	ADO84108	ADO84109	ADO84110	ADO84111
Ilyobacter polytropus DSM 2926 **(2)**	Fusobacteria	ADO83075	ADO83074	ADO83073	ADO83072	ADO83071
Klebsiella pneumoniae subsp. pneumoniae MGH 78578	Gamma-proteobacteria	YP_001337151	YP_001337150	YP_001337149	YP_001337148	YP_001337154
Lactobacillus reuteri DSM 20016	Firmicutes	ABQ83986	ABQ83985	YP_001272321	ABQ83983	YP_001272319
Listeria innocua Clip11262	Firmicutes	NP_470454	NP_470455	NP_470456	NP_470457	NP_470458
Listeria monocytogenes Clip81459	Firmicutes	YP_002757861	YP_002757862	YP_002757863	YP_002757864	YP_002757865
Mesorhizobium loti MAFF303099	Alpha-proteobacteria	NP_107174	Fused with *dhab1*	NP_107173	absent	absent
Mesorhizobium opportunistum LMG24607	Alpha-proteobacteria	AEH87445	Fused with *dhab1*	AEH87444	absent	absent
Mycobacterium smegmatis str. MC2 155	Actinobacteria	YP_885929	YP_885930	AFP37982	YP_890537	YP_885931
Pediococcus claussenii ATCC BAA-344	Firmicutes	AEV95608.1	AEV95607.1	AEV95606.1	AEV95605.1	AEV95598.1
Propionibacterium freudenreichii subsp. shermanii CIRM-BIA1	Actinobacteria	YP_003687866	YP_003687867	YP_003687868	YP_003687869	YP_003687870
Salmonella agona (strain SL483)	Gamma-proteobacteria	ACH51686.1	ACH48848.1	ACH50682.1	ACH48984.1	ACH51519.1
Salmonella enterica subsp. enterica serovar Weltevreden str. HI_N05-537	Gamma-proteobacteria	ZP_02830273	ZP_02830272	ZP_02830271	ZP_02830270	ZP_02830269
Salmonella typhimurium (strain LT2 / SGSC1412 / ATCC 700720)	Gamma-proteobacteria	AAL20944.1	AAL20945.1	AAL20946.1	AAL20947.1	AAL20954.1
Sebaldella termitidis ATCC 33386	Fusobacteria	YP_003307830	YP_003307831	YP_003307832	YP_003307833	YP_003307834
Shigella sonnei 53G	Gamma-proteobacteria	EFZ54557.1	EFZ54556.1	EFZ54555.1	EFZ54554.1	EFZ54547.1
Streptococcus sanguinis SK36	Firmicutes	YP_001034530	YP_001034531	YP_001034532	YP_001034533	YP_001034534
Thermoanaerobacter sp. X513	Firmicutes	ADN54603.1	ADN54604.1	ADN54605.1	ADN54606.1	ADN54607.1
Thermosediminibacter oceani DSM 16646	Firmicutes	YP_003824945	YP_003824946	YP_003824947	YP_003824948	YP_003824949
Tolumonas auensis (strain DSM 9187 / TA4)	Gamma-proteobacteria	ACQ93296.1	ACQ93297.1	ACQ93298.1	ACQ93299.1	ACQ93306.1
Veillonella dispar ATCC 17748	Firmicutes	ZP_04598907	ZP_04598908	ZP_04598909	ZP_04598910	ZP_04598911
Yersinia enterocolitica subsp. enterocolitica 8081	Gamma-proteobacteria	YP_001006921	YP_001006922	YP_001006923	YP_001006924	YP_001006925
